# Kaempferol pretreatment modulates systemic inflammation and oxidative stress following hemorrhagic shock in mice

**DOI:** 10.1186/s13020-015-0035-z

**Published:** 2015-03-21

**Authors:** Qi-Sheng Yang, Li-Ping He, Xian-Long Zhou, Yan Zhao, Jun Shen, Peng Xu, Shao-Zhou Ni

**Affiliations:** Emergency Center, Zhongnan Hospital of Wuhan University, Wuhan, Hubei 430071 China; Department of Anesthesiology, Tong Cheng Hospital, Tongcheng, Hubei 437400 China

**Keywords:** Hemorrhagic shock, Kaempferol, Oxidative stress, Systematic inflammation

## Abstract

**Background:**

Kaempferol has been reported as beneficial for both acute and chronic inflammatory diseases. This study aims to investigate whether kaempferol affects systemic inflammation and oxidative stress in the heart, lung, and liver after hemorrhagic shock in mice.

**Methods:**

Male C57/BL6 mice underwent hemorrhagic shock (mean arterial pressure of 35 mmHg for 90 min) and were arbitrarily divided into Sham, hemorrhagic shock (HS), and Kae groups (n = 10 in each group). Mice in the Kae groups received a kaempferol (10-mg/kg body weight) injection 12 h prior to (Group Kae PT) or 90 min after (Group Kae T) the initiation of hemorrhagic shock. Plasma proinflammatory cytokines (TNF-α and IL-6), organ myeloperoxidase (MPO) and superoxide dismutase (SOD) activities, and organ malondialdehyde (MDA) concentrations and heme oxygenase-1 (HO-1) expression levels were assessed by enzyme-linked immunosorbent assay (ELISA) or western blot assay.

**Results:**

Compared with the HS group and the Kae T group, pretreatment with kaempferol significantly decreased proinflammatory cytokines TNF-α (*P* = 0.012 and 0.015, respectively) and IL-6 (*P* = 0.023 and 0.014, respectively) following hemorrhagic shock. Kae pretreatment reverted MPO, SOD, and MDA to basal levels in the heart, lung, and liver (*P*s < 0.05), while the Kae T group showed no significant differences in these biomarkers compared with the HS group (*P*s > 0.05). HO-1 expression was significantly increased in the Kae PT group compared with the other groups (*P* = 0.011 *vs.* HS group and *P* = 0.02 *vs.* Kae T group).

**Conclusions:**

Pretreatment of hemorrhagic shock mice with kaempferol significantly decreased plasma levels of TNF-α and IL-6; reverted MPO, SOD, and MDA in the heart, lung, and liver; and increased expression of HO-1 in the same organs.

## Background

Hemorrhagic shock (HS) is one of the most common causes of deaths related to severe trauma [1,2]. HS is generally characterized by hemodynamic instability with cellular hypoxia and diminishing organ function [3]. Trauma and HS may stimulate a systemic release of endogenous molecules and provoke local and systemic liberation of both pro- and anti-inflammatory cytokines such as interleukin-1 beta (IL-1β), IL-2, IL-4, and tumor necrosis factor-alpha (TNF-α) [4,5]. Thus, severe and developing HS ultimately results in systematic inflammatory response syndrome or multiple organ failure and even death [6]. In addition, oxidative stress is implicated in the high mortality and morbidity of HS [6], and treatment with antioxidative stress agents could improve the outcome of HS [7-9].

Kaempferol is believed to have anti-inflammatory activities. It has been shown to have potential immune-modulatory effects and to interfere with a number of inflammation mechanisms *in vitro* [10,11]. Kowalski *et al.* [11] demonstrated that kaempferol blocks expression of both IL-1β and TNF-α in macrophages. Kaempferol significantly prohibits TNF assembly [12], and inhibits IL-2- and IL-4-mediated inflammatory outcomes [12,13]. Although both antioxidant and pro-oxidant activities of flavonoids have been previously studied [14,15], the antioxidant or pro-oxidant ability of kaempferol in HS has not been investigated.

This study aims to investigate whether kaempferol affects systematic inflammation and oxidative stress in the heart, lung, and liver after HS in mice.

## Methods and materials

### Animals

Forty 8–10-week-old specific pathogen-free male C57/BL6 mice weighing 20–25 g were purchased from the Center for Animal Experiment of Wuhan University (Wuhan, Hubei, China). The Research Committee of Wuhan University approved this study (No. 20130037). All experiments were conducted in accordance with the guidelines of the Animal Use and Care Committee of Wuhan University. Mice were housed individually in cages on a 12-h day/12-h night cycle at 23 − 27°C and had free access to food and water.

### Animal model

An HS model was used as previously described [16]. Briefly, animals were anesthetized with an intraperitoneal (i.p.) injection of pentobarbital sodium (50-mg/kg body weight [BW]; Amresco, Cleveland, OH, USA). Cannulation was performed using sterile polyethylene tubing on both right and left femoral arteries. All the catheters and syringes were pretreated with heparinized normal saline (25 IU/mL, Shuanghe, Beijing, China). The right femoral artery cannulation tube was connected with a pressure transducer to the BL-420 F biological signal acquisition system (Chengdu Taineng Ltd., Chengdu, China). Mean arterial pressure (MAP) was recorded throughout this experiment. HS was performed by withdrawing blood over a 15-min interval *via* the left femoral artery cannulation tube. MAP was controlled at 30–40 mmHg for 90 min followed by 15-min fluid resuscitation using an equal volume of normal saline as lost blood. Thereafter, the catheter was removed, the artery ligated, and the skin incision closed. After a recovery phase of 4.5 h, the mice were sacrificed. Blood samples, and heart, lung, and liver tissue were collected for biochemical analysis.

### Group distribution

**Figure 1:**
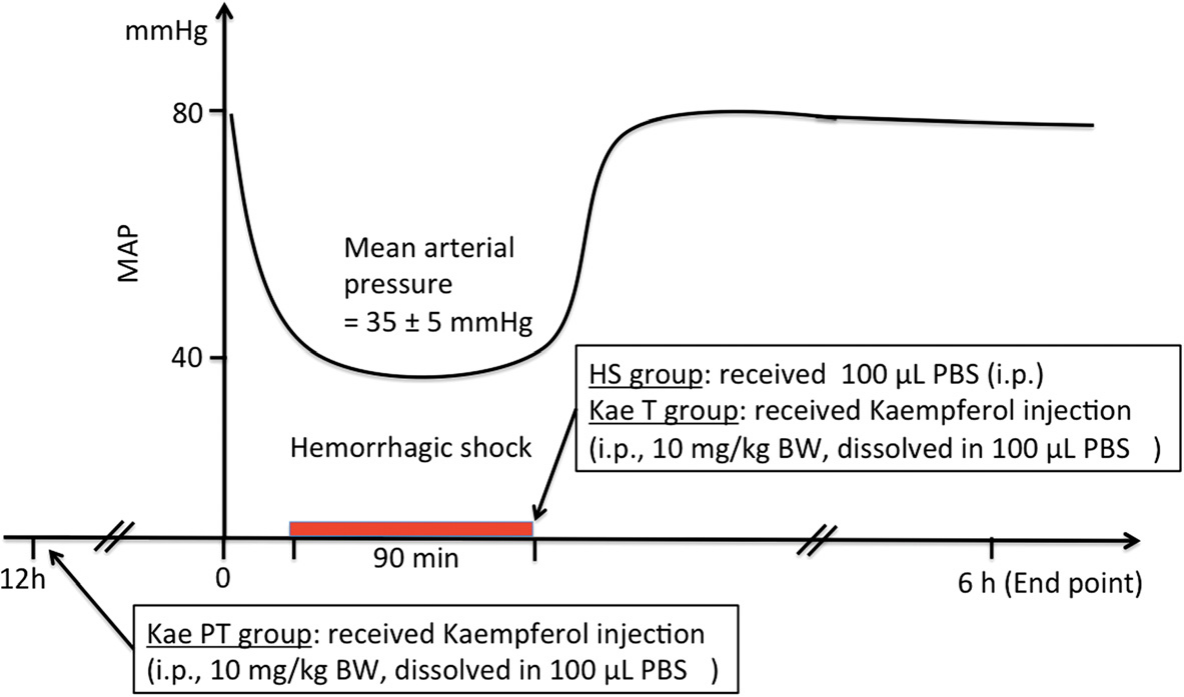
**Study protocol.** Animals were assigned to Sham (n = 10), HS (n = 10), Kae T (n = 10), and Kae PT (n = 10) groups. The HS group received an injection of 100-μL PBS (i.p.) at the end of the shock period. Kae T and Kae PT groups received an injection of kaempferol (i.p., 10 mg/kg BW, dissolved in 100-μL PBS) 90 min following or 12 h prior to the initiation of HS, respectively. Animals were sacrificed 6 h after the initiation of HS for blood and tissue sample collection.

Mice were arbitrarily divided into four groups: (1) a Sham group (n = 10): animals received femoral artery catheterizations without drawing blood; (2) a hemorrhagic shock (HS) group (n = 10): animals underwent HS and received an i.p. injection of 100-μL phosphate-buffered saline (PBS) (Boster, Wuhan, China) at the end of the 90-min shock period; (3) a kaempferol pretreatment (Kae PT) group (n = 10): animals received an injection of kaempferol (Sigma, St. Louis, MO, USA) (i.p., 10 mg/kg BW, dissolved in 100 μL of PBS) 12 h prior to the initiation of HS; and (4) a kaempferol treatment (Kae T) group (n = 10): HS animals received a kaempferol injection (i.p., 10 mg/kg BW in 100 μL of PBS) at the end of the shock period (Figure 1).

### Plasma TNF-α and IL-6 measurements

At the end of the study, blood samples were collected by cardiac puncture. Heparinized blood samples were centrifuged (5415D, Eppendorf, Germany) at 1200 × *g* for 10 min at 4°C. Thereafter, supernatants were stored at −80°C until measurements. Plasma cytokines including TNF-α (Cat no. MTA00B) and IL-6 (Cat no. M6000B) were measured by commercial enzyme-linked immunosorbent assay (ELISA) kits (R&D Systems Inc., Minneapolis, MN, USA) according to the manufacturer’s protocols.

### Myeloperoxidase activity

Fresh heart, lung, and liver tissues were frozen at the end of the study. Tissue was homogenized in a lysis buffer before the determination of myeloperoxidase (MPO) activity. MPO activity was determined by a commercial ELISA kit (Cat no. LS-F278-1, Biocompare, CA, USA) according to the manufacturer’s protocols. Previous studies [16,17] have reported observations of only minor histological changes following HS models. Therefore, we did not perform histological evaluation in this study.

### Malondialdehyde levels

Fresh tissue was placed into a 1.5-ml centrifuge tube. RIPA Buffer 250 μL was added with protease inhibitors. Homogenate was then centrifuged at 11000 × *g* for 10 min at 4°C. The supernatant was used for determining malondialdehyde (MDA) by a commercial kit (Cat no. 10009055, Cayman, Ann Arbor, MI).

### Superoxide dismutase activity

The supernatant of homogenate after centrifuge (12,000 × *g*, 10 min; 5415D, Eppendorf, Germany) was used for detecting superoxide dismutase (SOD) activity. Tissue SOD activity was measured with a SOD-525TM reagent kit (OXIS International, Foster, CA, USA); the final result was expressed as U/mg protein.

### Heme oxygenase-1 expression in organs

Western blot assay was used to detect the expression of heme oxygenase-1 (HO-1) in the main organs after HS. Briefly, 40-μg protein extracts were loaded onto a 10% resolving gel for electrophoresis. Proteins were transblotted onto membranes (Millipore, Temecula, CA, USA). Then, membranes were blocked for 1 h at room temperature. The blot was immune-probed overnight with primary antibodies including HO-1 (1:2000; Abcam, Cambridge, MA, USA) and β-actin (1:1000; Santa Cruz Biotechnology, Dallas, TX, USA). The blots were incubated with a secondary antibody for 1 h at room temperature. The signals were detected by enhanced chemiluminescene (ECL) and quantified by Photoshop CS6 software (Adobe, USA).

### Statistical analysis

Data were processed using the statistical analysis software SPSS version 18.0 (SPSS Inc., Chicago, IL, USA) and were expressed as mean ± SD. Comparisons of several means were performed using one-way and repeated measures two-way analysis of variance followed by the Tukey–Kramer test to identify significant differences between groups. All statistical significance tests were two-tailed and *P* values of less than 0.05 were considered significant.

## Results

### General characteristics of mice

The general characteristics of animals were shown in Table 1. The average BW and age were similar for all groups. MAP was recorded throughout the experiments. MAP during the HS period (MAP-in-HS) and after fluid resuscitation (MAP-post-HS) was calculated. MAP dramatically decreased after blood withdrawals, and MAP in the HS period was well controlled at 30–40 mmHg. There were no significant differences in MAP-in-HS and MAP-post-HS between the HS, Kae PT, and Kae T groups (HS *vs.* Kae PT, *P* = 0.14; Kae PT *vs.* Kae T, *P* = 0.32; HS *vs.* Kae T, *P* = 0.10). However, both MAP-in-HS and MAP-post-HS were significantly higher in the Sham group than in the HS, Kae PT, and Kae T groups (MAP-in-HS: *P* = 0.001, 0.002, and 0.001, respectively and MAP-post-HS: *P* = 0.012, 0.011, and 0.036, respectively).

**Table 1:** **General characteristics in all groups (mean ± SD)**

**Groups (n)**	**Age (d)**	**Body weight (g)**	**MAP-in-HS** ^**1**^ **(mmHg)**	**MAP-post-HS** ^**2**^ **(mmHg)**
Sham (10)	63 ± 5	23.0 ± 1.5	85.4 ± 7.5^##^	82.6 ± 6.4^#^
HS (10)	63 ± 5	22.0 ± 1.2	36.4 ± 3.0	74.5 ± 5.5
Kae PT (10)	64 ± 4	23.3 ± 1.5	35.0 ± 2.9	78.8 ± 6.0
Kae T (10)	64 ± 5	22.1 ± 2.4	36.2 ± 3.5	76.5 ± 7.0

^1^MAP-in-HS: MAP during a 90-min HS period; ^2^MAP-post-HS: MAP after normal saline resuscitation; MAP was automatically recorded and calculated *via* a BL-420 F biological signal acquisition system. No significant differences were found in age and body weight between groups. However, both MAP-in-HS and MAP-post-HS were significantly higher in the Sham group than in the HS, Kae PT, and Kae T groups. Compared with the other groups, ^#^ *P* < 0.05, ^##^ *P* < 0.01.

### Measurements of plasma cytokines

**Figure 2:**
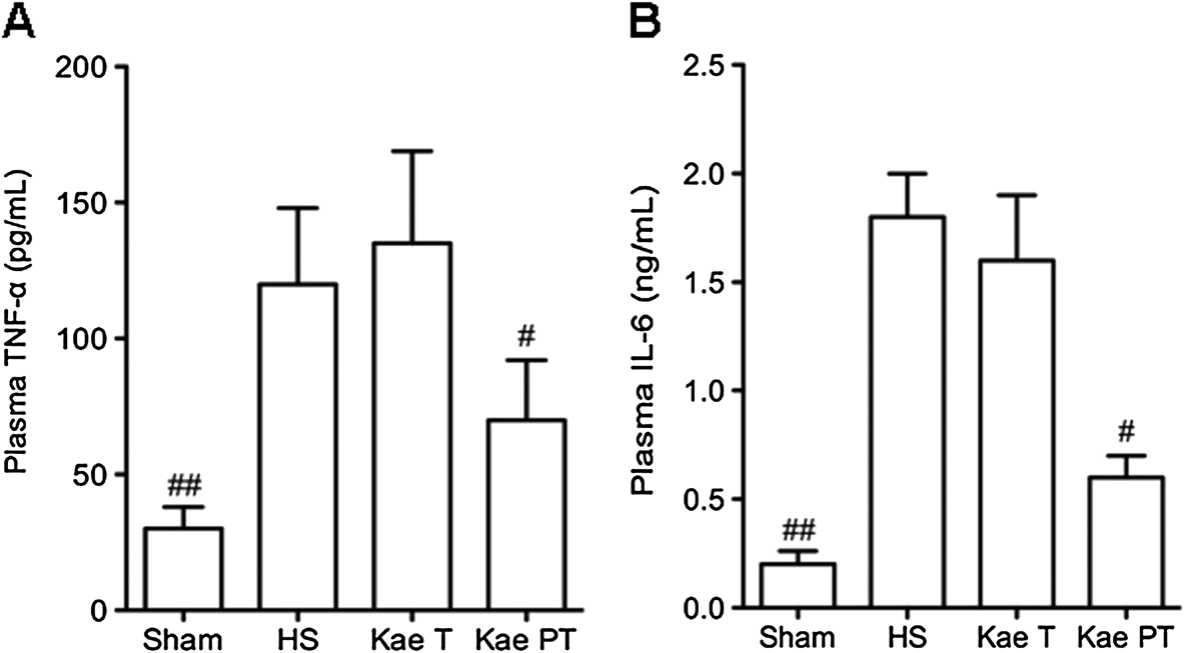
**Changes of plasma TNF-α (A) and IL-6 (B) in all groups.** Both TNF-α and IL-6 significantly increased after HS. Pretreatment with kaempferol reverted these two cytokines to basal levels. Compared with the other groups, ^#^ *P* < 0.05, ^##^ *P* < 0.01.

Compared with the Sham group, the plasma levels of TNF-α and IL-6 were significantly increased after HS in the HS (*P* = 0.001), Kae PT (*P* = 0.001), and Kae T (*P* = 0.0034) groups (Figure 2). The i.p. injection of kaempferol 12 h prior to the initiation of HS inhibited the release of both TNF-α (*P* = 0.012 *vs.* HS; *P* = 0.015 *vs.* Kae T) and IL-6 (*P* = 0.023 *vs.* HS; *P* = 0.014 *vs.* Kae T), while the injection of kaempferol 90 min following HS affected neither the plasma TNF-α nor the IL-6 levels.

### Organ inflammation

**Figure 3:**
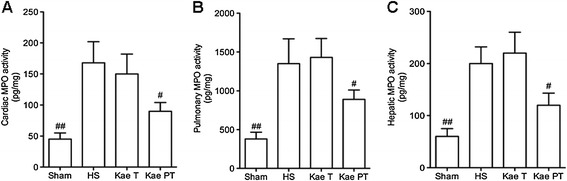
**MPO activities in heart (A), lung (B), and liver (C).** Elevated MPO activities were observed in the heart, lung, and liver after HS. MPO activity in the Kae PT group was significantly decreased compared with the HS and Kae T groups. Compared with the other groups, ^#^ *P* < 0.05, ^##^ *P* < 0.01.

The MPO activities were measured in the heart, lung, and liver (Figure 3). Elevated cardiac, pulmonary, and hepatic MPO activities were observed after HS in the HS, Kae PT, and Kae T groups. There was no significant difference in MPO activity between the HS and Kae T groups (*P* = 0.34, 0.19, and 0.074 in the heart, lung, and liver, respectively). However, the MPO activity was significantly decreased in the Kae PT group compared with the HS and Kae T groups (heart: *P* = 0.018 and 0.01; lung: *P* = 0.024 and 0.017; liver: *P* = 0.011 and 0.031, respectively).

### Organ oxidative response

**Figure 4:**
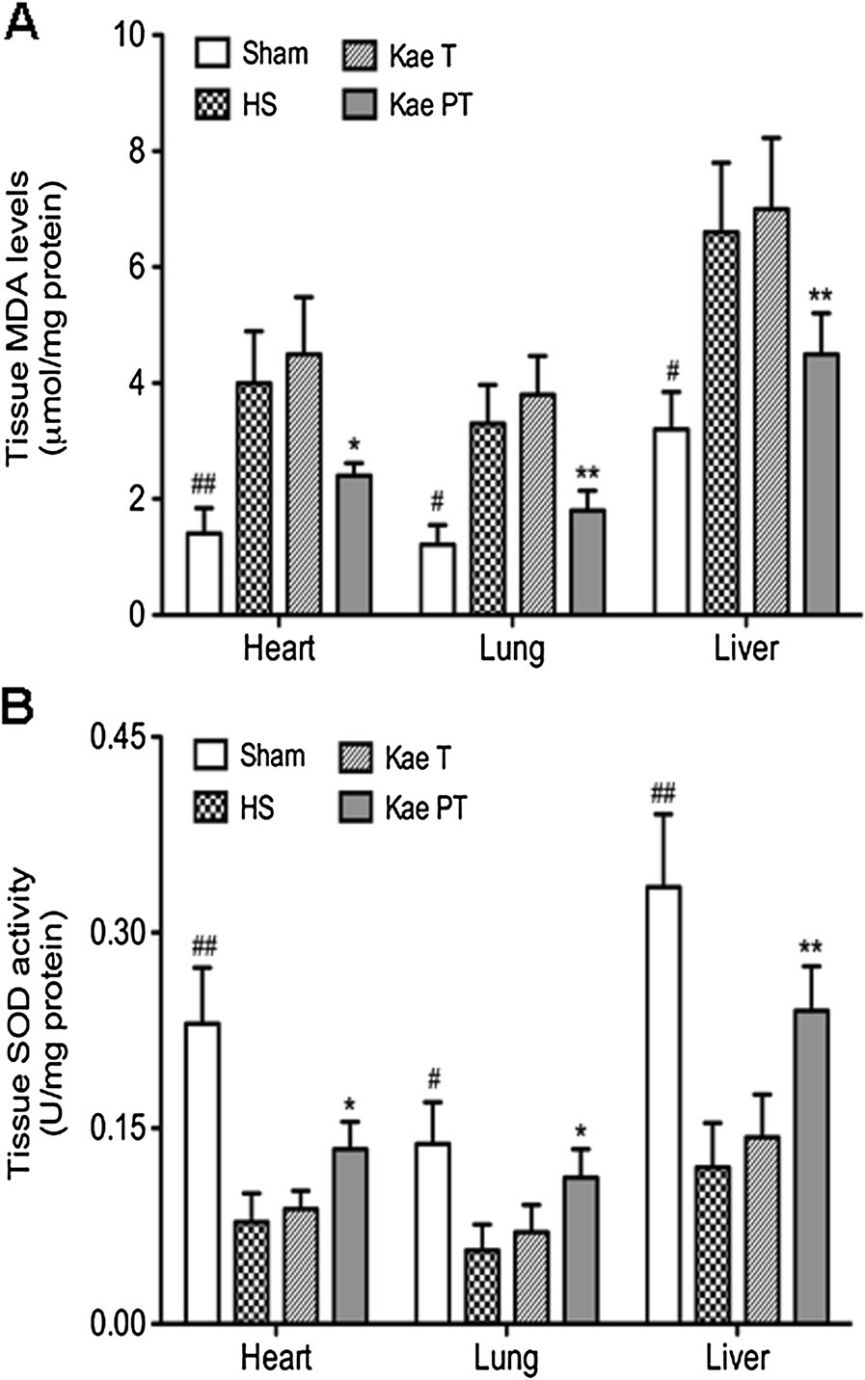
**Organ oxidative response after HS in all groups.** MDA levels **(A)** in the heart, lung, and liver were significantly increased, while SOD activities **(B)** were significantly decreased in all experimental groups after HS. Injection with kaempferol 12 h prior to the initiation of HS resulted in a significant decrease of MDA levels and increase of SOD activities. Compared with the other groups, ^#^ *P* < 0.05, ^##^ *P* < 0.01. Compared with the HS group or Kae T group, *P < 0.05, **P < 0.01.

We evaluated the MDA levels and SOD activities in the heart, lung, and liver to investigate the effects of kaempferol on oxidative response following HS. As seen in Figure 4, MDA levels were significantly higher in the HS groups than in the Sham group (*P* = 0.001, 0.016, and 0.011 in the heart, lung, and liver, respectively), while SOD activities were decreased in the HS groups compared with the Sham group (*P* = 0.001, 0.023, and 0.010 in the heart, lung, and liver, respectively). The injection of kaempferol following HS (Kae T) showed no effects on MDA levels in the heart (*P* = 0.34), lung (*P* = 0.43), and liver (*P* = 0.11) and on SOD activities in the heart (*P* = 0.16), lung (*P* = 0.37), and liver (*P* = 0.25) compared with the HS group. However, the injection of kaempferol 12 h prior to the induction of HS resulted in decreased MDA levels (*P* = 0.012, 0.001, and 0.002, respectively) and increased SOD activities (*P* = 0.032, 0.024, and 0.004, respectively) compared with the HS group in the heart, lung, and liver.

### Expression of HO-1 in the heart, lung, and liver

**Figure 5:**
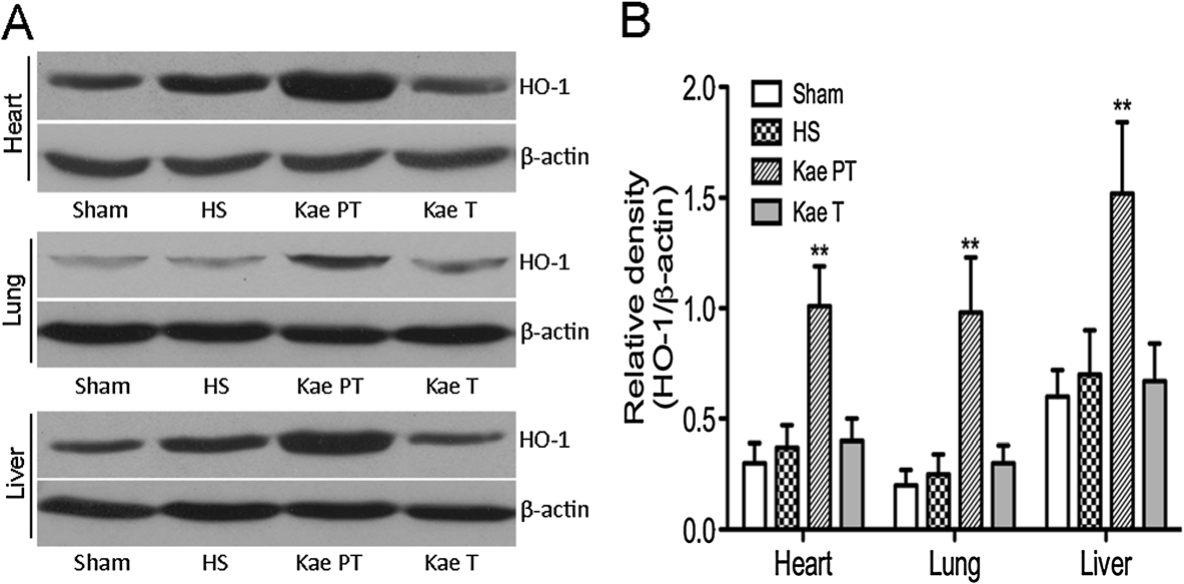
**HO-1 expression in heart, lung, and liver after HS. A**, Western blot assay for HO-1 expression; **B**, relative density (HO-1/β-actin). Injection with kaempferol 12 h prior to the initiation of HS resulted in a significant increase of HO-1 expression in the heart, lung, and liver. Compared with the other groups, **P < 0.01.

This study found evidence of HO-1 expression in the main organs including the heart, lung, and liver. As seen in Figure 5, HO-1 expression was insignificantly increased in the heart, lung, and liver in the HS group compared with the Sham group (*P* = 0.37, 0.22, and 0.19, respectively). The injection of kaempferol following HS showed no effects on HO-1 expression in organs including the heart (*P* = 0.063), lung (*P* = 0.21), and liver (*P* = 0.37) after HS, while the injection of kaempferol 12 h prior to the induction of HS resulted in significantly increased expression of HO-1 in the heart (*P* < 0.001), lung (*P* = 0.001), and liver (*P* < 0.001) compared with the HS group.

## Discussion

In this study, we investigated the protective effects of kaempferol in HS in mice. The injection of kaempferol prior to the initiation of HS significantly decreased serum proinflammatory cytokines and organ inflammation. Moreover, kaempferol showed its antioxidant ability through inhibiting MDA expression and promoting SOD activities in the heart, lung, and liver.

HS and subsequent ischemia/reperfusion injury are associated with oxidative stress in organs [18,19], and it has been proposed that this generated oxidative stress is a fundamental mechanism of organ damage in HS [20]. Oxygen-free radicals are involved in HS and subsequent resuscitation [21]. Wu *et al.* [22] confirmed that HS and resuscitation induced significant oxidation in rat lungs, while Panteli *et al.* [23] reported that the oxidative state of the kidneys remained unaffected after HS. Treatment with antioxidant agents after HS reduced organ oxidative stress and led to a better outcome. Antioxidant therapy with Tempol, a membrane-permeable radical scavenger, improved outcome after HS in rats [24]*.* In our study, HS and resuscitation induced significant oxidative stress in the heart, lung, and liver, but pretreatment with kaempferol decreased oxidative stress in these organs.

Oxidative stress in HS induces the activation of nuclear factor-κB (NF-κB), which triggers an inflammatory cascade during acute hemorrhage, and leads to an overproduction of proinflammatory cytokines [25-27]. Moreover, oxidative stress during HS can induce accumulation and activation of neutrophils and subsequent increases in concentrations of inflammatory mediators, including TNF-α and IL-6, in organs [28,29]. The TNF-α and IL-6 plasma levels were significantly increased after HS in our study. In addition, the i.p. injection of kaempferol at a single dose 12 h prior to the initiation of HS was associated with significantly decreased organ inflammation. This finding was possibly a consequence of the reduced systemic inflammatory response observed in our experiment. One previous study found that the activation of NF-κB could be attenuated by treatment with kaempferol [30]. Therefore, its anti-inflammatory and antioxidant abilities in this study were possibly associated with the inhibition of NF-κB expressions after HS.

Modulation of HO-1, also known as heat shock protein 32, is a promising approach to the attenuation of organ damage [31]. HO-1 is a stress-inducible protein, and its overexpression protects against ischemia-reperfusion injury [32]. Up-regulation of HO-1 could lead to remarkable cytoprotective effects and attenuate organ damage after HS [33]. In addition, kaempferol protected cells against apoptosis *via* inductions of HO-1 expression [34]. In this study, kaempferol pretreatment resulted in significantly higher levels of HO-1 in major organs including the heart, lung, and liver. Future studies are needed to investigate whether kaempferol also exerts its protective effects *via* the up-regulation of HO-1 in tissues after HS.

## Conclusions

Pretreatment with kaempferol of HS mice significantly decreased plasma levels of TNF-α and IL-6; reverted MPO, SOD, and MDA in the heart, lung, and liver; and increased expression of HO-1 in the same organs.

## Abbreviations

BW: Body weight
ECL: Enhanced chemiluminescene
ELISA: Enzyme-linked immunosorbent assay
HS: Hemorrhagic shock
HO-1: Heme oxygenase-1
IL-1β: Interleukin-1 beta
IP: Intraperitoneal
MAP: Mean arterial pressure
MDA: Malondialdehyde
MPO: Myeloperoxidase
NF-κB: Nuclear factor-kappa B
PBS: Phosphate-buffered saline
SOD: Superoxide dismutase
TNF-α: Tumor necrosis factor-alpha
